# Narrowing the Skills Gap for Innovation: An Empirical Study in the Hospital Sector

**DOI:** 10.2196/humanfactors.3598

**Published:** 2014-09-23

**Authors:** Casimiro Dias, Ana Escoval

**Affiliations:** ^1^ World Health Organization Health Systems and Public Health Copenhagen Denmark; ^2^ University Nova Lisboa School of Public Health Lisbon Portugal

**Keywords:** hospital administration, organizational innovation, management information systems, clinical skills, staff development, personnel management

## Abstract

**Background:**

The current financial crisis and the increasing burden of chronic diseases are challenging hospitals to enhance their innovation capacity to deliver new and more effective health services. However, the shortage of skills has been widely recognized as a key obstacle for innovation. Ensuring the presence of a skilled workforce has become a priority for the health system in Portugal and across Europe.

**Objective:**

The aim of this study was to examine the demand of new skills and their influence in both investments in innovation and development of skills.

**Methods:**

We used a mixed-methods approach combining statistical analysis of data survey and content analysis of semistructured interviews with the Administration Boards of hospitals, using a nominal group technique.

**Results:**

The results illustrate an increasing demand of a broad range of skills for innovation development, including responsibility and quality consciousness (with a significant increase of 55%, 52/95), adaptation skills (with an increase of 44%, 42/95) and cooperation and communication skills (with an increase of 55%, 52/95). Investments in the development of skills for innovation are mainly focused on aligning professional training with an organizational strategy (69%, 66/95) as well as collaboration in taskforces (61%, 58/95) and cross-department teams (60%, 57/95). However, the dynamics between the supply and demand of skills for innovation are better explained through a broader perspective of organizational changes towards enhancing learning opportunities and engagement of health professionals to boost innovation.

**Conclusions:**

The results of this study illustrate that hospitals are unlikely to enhance their innovation capacity if they pursue strategies failing to match the skills needed. Within this context, hospitals with high investments in innovation tend to invest more in skills development. The demand of skills and investments in training are influenced by many other factors, including the hospital’s strategies, as well as changes in the work organization. Relevant implications for managers and policy makers can be drawn from the empirical findings of this paper, building on the current efforts from leading innovating hospitals that are already defining the future of health care.

## Introduction

The increasing burden of chronic diseases, the aging population, and the current financial crisis are challenging hospitals to reinvent their health care delivery models, restructure their organizations, and redefine their value. Tackling such challenges require new skills for enhancing organizational flexibility, improving performance, and promoting innovation [1,2].

The shortage of skills remains a key obstacle for innovation, particularly in hospitals pursuing radical and disruptive changes. Innovation depends largely on the health professionals who are able to create and apply new ideas and knowledge in their daily work [3,4]. Therefore, it has become important to ensure that a skilled workforce is present for delivering high quality health services to strengthen the health system in Portugal, as well as across Europe. Such emphasis on skills and innovation is reflected by several efforts to bridge and narrow the current skills and productivity gap [5,6].

Hospitals have long focused on the development of skilled health professionals through continuous efforts in training and education. As they struggle to ensure the right skills for innovation, it is particularly relevant to better understand the different types of skills needed for innovation and the best ways to ensure continuous improvement.

However, previous efforts to make explicit the links between specific skills and innovation have proven difficult [7-9]. The broad definitions of skills and innovation, as well as the difficulty of measuring them have limited the identification of such relationships [10,11].

Previous research has been focused on the demand of skills to make the best use of the new technologies, while the investments in skill development by hospitals has been significantly less studied [12]. However, such narrow approach might result in several drawbacks, including an increased number of unsuitably qualified professionals or discouraging their engagement in innovation activities.

Recognizing the relevance of the dynamics between skills and innovation, current initiatives from the European Commission and Organisation for Economic Co-operation and Development (OECD) are supporting significant advances in this area. The New Skills for New Jobs initiative from the European Commission sets out to promote better anticipation of future skills needs, to reach a better match between skills and labour market needs as well as to bridge the gap between education sector and the labour market [13].

Empirical evidence suggests that the right combination of technological and organizational innovations may result in high relevance of interpersonal competencies or so called “people skills” as well as non-routine problem-solving [14,15].

Furthermore, OECD Skills Strategy provides an integrated strategic framework to better understand effective ways of skills development for economic growth. The strategy takes a whole-government approach in order to match the supply and demand of skills for innovation [16]. The global independent commission on health professional education for the 21st century has proposed a strategic framework on health professional’s education for the next century with the objective of reaching a balance between population health needs, health-system demand for skills, and what the educational system can offer [17,18]. The Commission proposes a new era of health professional education is mainly based on transformative learning and interdependence in education. All these initiatives highlight that the future will be based on adjustments of skills to specific contexts, which draws on the power of flows of information and knowledge.

This paper takes a dynamic approach with a focus on the changing trends with specific definitions in three dimensions. Technological innovation refers to the development and dissemination of new technologies, including information and communication technologies (ICT). Organizational innovation refers to changes in the work organization including investments in new skills and competences. Finally, skill changes reflect the combination of these factors, thus redefining the skills within new organizational processes. Such trends are particularly relevant to better understand how the hospital sector is being reshaped to improve innovation capacity. Empirical evidence suggests that the right combination of technological and organizational innovations may result in high relevance of interpersonal competencies, as well as non-routine problem solving [19,20].

The aim of this paper is to explore the relationship between skills and innovation. It analyses the interaction between supply and demand for skills within a dynamic approach, such that both innovation and skill requirements are also changed. Throughout the hospital sector, there are several forward-thinking hospitals, which are developing new skills for enhancing their innovation capacity. Therefore, we will further explore the way innovating hospitals are already matching both skills and innovation development.

## Methods

### Overview

A mixed methods design was used to explore the relationship between skills and innovation development at the hospital sector. This study systematically integrates multiple forms of quantitative and qualitative data collected through a survey, a series of interviews and a technical nominal group.

### Demand of Skills and Work Organization

Primary data was collected through a survey in order to examine the changes in the skills supply and demand, and their impact on innovation capacity of hospitals. A revised version of the survey Danish System of Innovation in a Comparative Perspective (DISKO) developed by the Danish Research Unit for Industrial Dynamics (DRUID), was used and adapted to the hospital sector [21]. This survey is based on the search for “organizational traits” related to the hospital capacity to adapt changing and unstable environments. The survey measures the changes in terms of skills, work organization, as well as innovation capacity of hospitals during a period of 3 years since 2007. The survey was submitted to a pre-test in 6 different hospitals to assess its validity, reliability, and comprehensibility.

The paper-survey was submitted by post mail to a national sample of 136 hospital boards, identified from the official list of hospitals in the Portuguese public sector. A total number of 95 administrators from hospital management boards replied to the survey during a period of three months, corresponding to a response rate of 70%.

Statistical analysis of the data was performed in order to examine the current trends for the skills needed for innovation in the hospital sector. In all cases, the level of significance was *P*<.05. All statistics were calculated with SPSS version 15.0 (SPSS Inc, Chicago, IL, USA).

### Development of Skills for Innovation

A series of interviews were carried out in 5 hospitals, which were selected based on the highest levels of investments in both innovation and skills development, according to the survey data. Semistructured and face-to-face interviews were undertaken with the aim of identifying the major mechanisms for matching skills and innovation development at the hospital sector.

The data from the interviews was submitted for content analysis, which consisted of a comparative assessment of the frequency and significance of the different categories.

### Dynamics Between Skills and Innovation Development

Finally, the nominal group technique was applied to the data with the objective of consensualizing the major findings in terms of the relationships between skills and innovation development. The group included experts from hospital administration, financing, information system, and human resources development. A modified nominal group technique was applied by introducing evidence for discussion in a stepwise way before voting. The use of votes by the experts was to overcome an unequal representation of different opinions.

The analysis of both qualitative and quantitative data has brought a comprehensive picture of the interaction between skills and innovation at the hospital level, which might have been overlooked by a simpler method.

## Results

### Overview

The results revealed the main trends of the skills demand, as well as their influence in the innovation capacity of hospitals. It further explores the complementarities between skills and innovation, as well as major mechanisms for advancing both at the hospital level.

### Demand of Skills and Organizational Innovation

The administration boards of hospitals were explicitly asked about changes in the demand for skills during a period of three years. The demand of skills was analysed in four dimensions: responsibility and quality consciousness, ability of adaptation to change, cooperation and communication skills, as well as vocational qualifications.

Table 1 shows an increasing demand of a broad range of “generic” skills, including responsibility and quality consciousness (52/95, 55%), adaptation (42/95, 44%), and ability to cooperate and communicate (52/95, 55%).

However, there were significant differences in terms of the skills needed according to the innovation capacity of hospital. Therefore, the total number of hospitals was clustered by the innovation rate, which was measured as the number of innovations developed by the hospital per year. The two clusters by innovation capacity included 38 innovating and 57 non-innovating hospitals.

However, innovating hospitals revealed a significantly higher demand of skills for innovation, including a 72% (28/38) increase in the demand for responsibility and quality consciousness (**P*=.*02), and 63% (24/38) increase for adaptation skills (*P*=.03), as well as an increase of 62% (24/38) for communication and cooperation skills (*P*=.02).

**Table 1:** Number of hospitals with increasing demand of specific skills in a three years period.

Skills	Innovating hospitals (n=38)	Non-innovating hospitals (n=57)	All (N=95)
	n (%)	n (%)	n (%)
Responsibility and quality consciousness	28 (72)	24 (42)	52 (55)
Adaptation	24 (63)	18 (32)	42 (44)
Communication and cooperation	24 (62)	28 (32)	52 (55)
Vocational qualifications	21 (56)	18 (31)	39 (41)

Table 2 shows the main changes of work organization, which are related to the development of skills in the hospital sector. These new forms of work organization are mainly focused on the increase of delegation of tasks by 21% (20/95), transversal work groups by 13% (12/95), integrated services delivery by 14% (13/95), as well as quality circles, which has increased by 8% (8/95). Other organizational changes including remuneration based on performance (5/95, 5%), systems of gathering proposals from health professionals (2/95, 2%), and planned work rotation (5/95, 5%) are significantly less widespread in the hospitals.

The increasing use of these new forms of work organization is significantly higher in innovating hospitals. This is particularly true regarding the 22% (9/38) increase of transversal working groups (**P*=.*01), a 17% (6/38) increase of quality circles and services integration (*P*=.04), as well as a 14% (5/38) increase for remuneration based in performance (*P*=.01).

**Table 2:** Number of hospitals with an increasing adoption and use of new forms of work organization in a three-year period.

.	Innovating hospitals (n=38)	Non-innovating hospitals (n=57)	All (N=95)
	n (%)	n (%)	n (%)
Transversal work groups	9 (22)	3 (5)	12 (13)
Quality circles	6 (17)	2 (3)	8 (8)
Systems of gathering proposals from health professionals	2 (5)	0 (0)	2 (2)
Planned work rotation	2 (6)	3 (5)	5 (5)
Delegation of functions	15 (39)	5 (9)	20 (21)
Integrated services delivery	7 (17)	6 (10)	13 (14)
Remuneration based in performance	5 (14)	0 (0)	5 (5)

The survey data brings further insights in terms of the main drivers behind these trends. Such changes are mainly driven by the need to establish better contacts with users (52/95, 55%) and the introduction of new technologies (47/95, 49%). In less extension, they are also motivated by demands from health professionals (31/95, 35%), opportunities for skill development (31/95, 33%), need to establish better contacts with subcontractors (31/95, 33%), as well as the need for higher work flexibility for health professionals (31/95, 33%). Other drivers like competition (25/95, 26%) and the development of new products or services (23/95, 24%) are the least relevant to explain such trends in work organization.

### Development of Skills for Innovation

Given the increasing demand of skills for innovation, we further examined the way and the extent in which hospitals invest in skills development.

The majority of hospitals consider investments in skills development as key for innovation. However, the main differences lie in specific mechanisms of skills development for innovation. Table 3 shows the different use degree of mechanisms for the development of skills in the hospital sector.

Hospitals recognize the need to align professional training to the organization needs, as used in high extension by 63% (66/95) of the total number of hospitals. Other major mechanisms include the organization by working teams (58/95, 55%), as well as cross-department cooperation and long term planning of professional training, both extensively used by 54% (57/95) of hospitals.

**Table 3:** Number of hospitals using specific mechanisms for the development of skills in different extension degrees (N=95).

	High	Medium	Low	None
Professional training based on organizational needs	66 (63)	22 (21)	6 (6)	1 (1)
Organization by working teams	58 (55)	25 (24)	8 (8)	0 (0)
Cross- department cooperation	57 (54)	27 (24)	7 (7)	4 (4)
Long term plans for professional training	57(54)	27 (26)	4 (4)	6 (6)
Standard training courses	37 (35)	35 (33)	23 (22)	0 (0)
Time available for learning with other professionals	27 (26)	51 (49)	16 (15)	1 (1)
Planned job rotation	26 (25)	43 (41)	24 (23)	2 (2)

### Dynamics Between Skills and Innovation Development

The content analysis of interviews highlights the dynamic nature of skills and innovation development and the relevance of matching both processes.

The interviews to the administration boards of hospitals revealed that these hospitals are looking for the necessary skills in order to realize their strategy over the next years. Daily problem solving is preferred as a major mechanism to ensure the right skills for innovation by 32% (30/95) of the hospitals rather than traditional standard training, as referred by 26% (25/95) of the hospitals.

However, external cooperation is the key mechanism for matching the demand and supply of skills for innovation as mentioned by 42% (40/95) of the hospitals. The relevance role of external cooperation has been revealed both by the content analysis of the interviews as well as the expert panel, since it was not included in the survey.

Through the nominal group technique, the expert panel has agreed on the main priorities for matching match innovation and skills development at the hospital level. The expert panel has identified the development of integrated information systems as the top priority for sharing information and creating knowledge to boost organizational learning towards innovation. Other long-term measures included dynamic planning of human resources and streamlining the management of human resources. Furthermore, it was identified that management by objectives and professional development were “quick-win” measures. These priority measures reflect the key role of human resources management and planning in order to advance innovation capacity of hospitals.

## Discussion

### Demand of Skills and Organizational Innovation

Our results suggest that a broad range of skills is engaged in innovation development in the hospital sector. These include not only specific technical skills but also higher-level problem solving skills. In fact, innovation encompasses a broad range of activities, requiring the engagement of many different groups of health professionals. Such change relies largely on learning by doing while designing and implementing innovations in the hospital sector. Therefore, the so-called “generic” skills, including responsibility and quality consciousness, capacity to adapt, and communication and cooperation are particularly relevant for innovation development.

The results also suggest that organizational changes are a key factor for explaining the current demand of skills. New forms of work organization are mainly aimed at enhancing organizational flexibility. These measures include decentralization of management mechanisms, as well as dissemination of teamwork across the organization. This is particularly true to innovating hospitals, which tend to be highly collaborative with an emphasis on multifunctional teams engaged in problem-solving cycles supported by spaces for constructive dialogue. Therefore, health professionals must be able to take on different tasks and responsibilities through effective cooperation across hospital departments and other organizations. Rather than specific skills to perform standardized and isolated tasks, knowledge of the wider process in which they are involved is preferred.

### Development of Skills for Innovation

Hospitals are looking for highly skilled health professionals who are able to use and improve new technologies and services. Results suggest that hospitals with higher investments in innovation are also more willing to invest in skill development. Innovating hospitals differ especially in their demand for flexibility and communication abilities.

Increasing investments in skills development focus on informal ways of learning across the hospital’s departments, for example, through learning-by-doing and teamwork, rather than the traditional standard courses.

Both the hospital administration boards and the expert panel highlighted the transformative role of ICT in the hospital sector. The main argument for connecting investments in the development of skills and ICT is that skilled health professionals are able to ensure higher performance though the use of these technologies. However, the transformation of the health sector is probably better understood through a broader perspective of changes in the work organization.

Organizational innovations put a premium on the learning capability of hospitals to absorb change, as well as of the skills of interaction and communication. Such results points out the importance for supporting the creation of “learning organizations”. Within this context, investments in skills development further improve the innovative potential of hospitals by valuing the creative efforts and multiplying opportunities for learning across the hospital.

### Dynamics Between Skills and Innovation Development

Innovating hospitals pursue high value-added strategies, which typically involve strong emphasis on research, high technologies, and skill-intensive work. In many hospitals, financial incentives encourage short-term efficiency but fail to recognize the importance of experimentation to achieve innovation and greater efficiency. For example, some innovating hospitals allow their health professionals to spend one-sixth of their time on any project that interests them.

These hospitals also encourage collaborative arrangements with other organizations in order to gain long-term advantages from interactive learning, even if there are additional short-term costs. This is best achieved by confronting hospitals not with a single best practice model, but rather by encouraging a stepwise transformation. While such an approach might not be applicable to all hospitals, it has been proven to enhance health professionals’ commitment and motivation to boost innovation.

External cooperation with other organizations, for example, universities and research institutes, is another key mechanism. The way hospitals are engaging in these collaborative networks is critical for ensuring the right skills for innovation by sharing ideas and information, as well as redefining and reinventing their value.

The coordination of processes and collaboration among the broad range of stakeholders requires the capacity for monitoring and evaluating performance, including the value of interventions along the health care continuum, as well as the health outcomes for the patient measured over their lifetime. Innovating hospitals are already enhancing smart decision-making through the development of integrated information systems at the point of care. The capacity of hospitals to collect and store, use, and share information becomes a critical skill for innovation strategies. These new skills have the potential to transform the hospital sector by shifting from reactively treating diseases to a more proactive approach of health promotion. It is within this context that the knowledge created across the hospital sector can be translated into practice more effectively, with health professionals, researchers, and informatics collaborating in real time.

### Conclusions

The paper reveals the difficulty to disentangle the skills driving innovation from those as a result of change. The relationship between skills and innovation becomes circular and cumulative overtime.

A broad range of workforce skills are involved in the development of innovation in the hospital sector. This is particularly relevant in the hospital sector where incremental change in products, services, and processes remains as the predominant form of innovation. However, as most notably demonstrated in this paper, skills are a necessary but not sufficient condition for successful innovation. The demand of skills and investments in training are influenced by many other factors, including the hospital’s strategies, as well as changes in the work organization.

Empirical findings of this paper have relevant implications for managers and policymakers. While many decisions in terms of human resources remain at the core of the hospital management, national policies also have significant scope to influence such decisions. In particular, policies enabling organizational flexibility and facilitating investments in training may support hospitals in their innovation efforts. However, current policies have remained mainly as a top-down, fragmented approach, with a particular focus on the supply of skills for innovation. There is the need to adopt a more dynamic approach to reflect the interactions between innovation and skills. The development of such a comprehensive strategy for skills development, well aligned with organizational changes, might expand the set of potentially successful innovators in the hospital sector.

Three main policy options should be jointly taken, including the development of flexible learning schemes, strengthening cooperation between universities and hospitals, as well as the integration of information systems throughout the hospital sector. Besides the initial university education, health professionals need to continuously update their knowledge and upgrade their skills. Such training at the workplace builds work-related competencies and support health professionals to deal with change. The external cooperation with other stakeholders from the health, education, and research sectors are increasingly critical to improve the innovation capacity of hospitals. This can be mainly facilitated through an integrated information system across the different stakeholders engaged in the innovation process. However, policies for skills’ development further needs to be coherent and provide a supportive environment for innovation by enabling health professionals to develop the right skills and support their optimal use at work.

These policy and management implications should build on current efforts from innovating hospitals, which are already matching skills and innovation, as identified in this study. They are mainly focusing on person-centered health care, partnering with a broad range of stakeholders outside the traditional borders, and integrating information across the hospital sector in order to support smart decision-making. These innovating and high performing hospitals are already defining the future of health care.

## Abbreviations

DISKO: Danish System of Innovation in a Comparative Perspective
DRUID: Danish Research Unit for Industrial Dynamics
ICT: information and communication technologies
OECD: Organisation for Economic Co-operation and Development
